# EXPLORING DIGITAL LITERACY, HEALTH LITERACY, AND SELF-MANAGEMENT IN YOUNGER AND OLDER LIVER TRANSPLANT RECIPIENTS

**DOI:** 10.1093/geroni/igae098.2608

**Published:** 2024-12-31

**Authors:** Hanna Lee, JiYeon Choi, Seongmi Choi, Sang Hui Chu

**Affiliations:** Yonsei University College of Nursing, Mo-Im Kim Nursing Research Institute, Seoul, Republic of Korea; Yonsei University College of Nursing, Mo-Im Kim Nursing Research Institute, Seoul, Republic of Korea; Yonsei University College of Nursing, Mo-Im Kim Nursing Research Institute, Seoul, Republic of Korea; Yonsei University College of Nursing, Mo-Im Kim Nursing Research Institute, Seoul, Republic of Korea

## Abstract

Liver transplant recipients (LTRs) face challenges in self-management, including adhering to immunosuppressant therapy and navigating healthcare systems. Adequate health literacy enables LTRs to manage daily tasks, while digital proficiency enhances self-management. Understanding health and digital literacy among older LTRs is crucial for targeted self-management strategies amidst increasing digitalization and liver transplantation in older adults. This descriptive study compared health literacy, digital literacy, and self-management between younger LTRs (< 60 years; n=97, 50.88±7.19 years, 27 male, 20.57±17.42 months since LT) and older LTRs (≥ 60 years; n=58, 64.52±3.48 years, 21 male, 27.33±16.21 months since LT) and explored associations among these variables. LTRs responded to a cross-sectional survey on digital literacy (Everyday Digital Literacy Questionnaire, 22 items), health literacy (European Health Literacy Survey, 16 items), and self-management (modified Transplant Self-Management Scale, 16 items). Younger and older LTRs differed in self-management (56.95±9.43 vs 61.83±7.73, p< 0.001) and digital literacy (85.58±16.99 vs 71.17±22.98, p< 0.001), but not health literacy (13.62±3.43 vs 13.50±3.30, p=0.833). When exploring the role of health literacy and digital literacy in predicting self-management in the entire sample after controlling for age, sex, education level, household income, and time since LT, health literacy had a significant association with self-management (B=0.479, p=0.04) whereas digital literacy did not (B=0.007, p=0.87). In our sample, older LTRs reported better self-management and similar health literacy levels but poorer digital literacy compared to younger LTRs. Supporting digital literacy in older LTRs may enhance their self-management. Further studies are needed to understand digital literacy and self-management in older LTRs.

